# Supplementary material for: Streamlined analysis of drug targets by proteome integral solubility alteration indicates organ-specific engagement
Source: Nat Commun. 2024 Oct 16;15:8923. doi: 10.1038/s41467-024-53240-2 (PMC11484808; doi:10.1038/s41467-024-53240-2)
Supplement: Supplementary file 1 — Supplementary information [file 41467_2024_53240_MOESM1_ESM.pdf]

SUPPLEMENTARY INFORMATION FOR STREAMLINED ANALYSIS OF DRUG TARGETS BY PROTEOME INTEGRAL SOLUBILITY ALTERATION INDICATES ORGAN-SPECIFIC ENGAGEMENT

Tanveer Singh Batth[1], Marie Locard-Paulet[1,2] , Nadezhda T. Doncheva[1], Blanca Lopez Mendez[1], Lars Juhl Jensen[1], and Jesper Velgaard Olsen[1]

1) The Novo Nordisk Foundation Center for Protein Research, University of Copenhagen, Denmark
2) Current affiliation: Institut de Pharmacologie et de Biologie Structurale (IPBS), Université de Toulouse, CNRS, Université Toulouse III - Paul Sabatier (UT3), Toulouse, France

Supplementary Figure 1: TPP melting point distributions

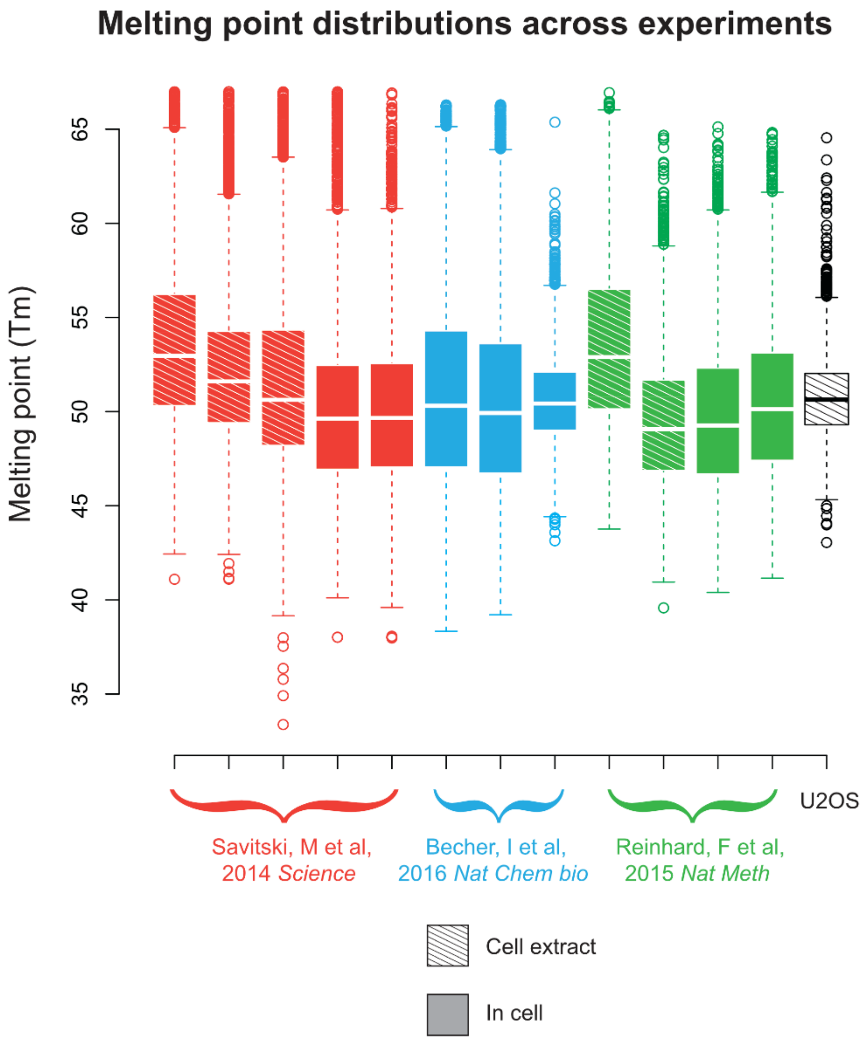

Supplementary Figure 1: Published melting point box plot distributions from different Thermal Proteome Profiling (TPP) experiments[1–3] are plotted including from U2OS generated from this study.

Supplementary Figure 2: Comparison between DIA and TMT for TPP-PISA experiments

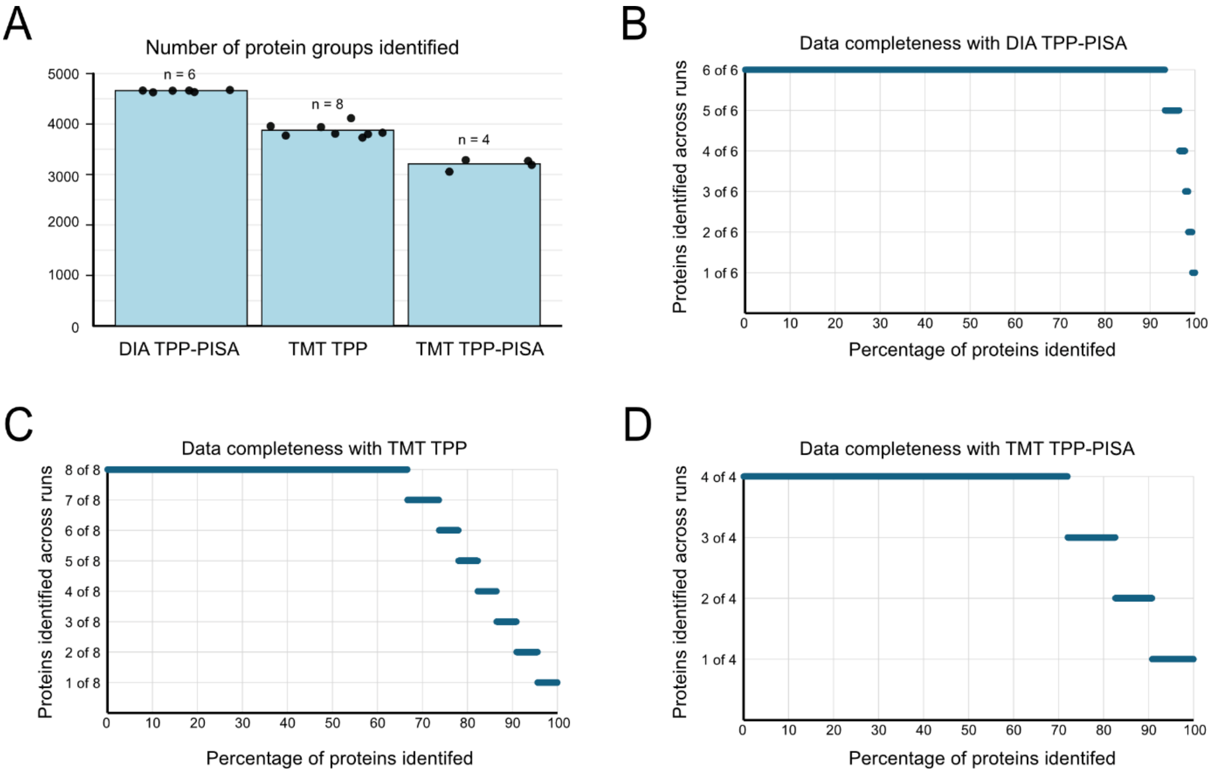

Supplementary Figure 2: Evaluation of protein identification rates and data completeness for TPP-PISA experiments using TMT labeling approach (DDA) vs DIA mass spectrometry analysis. A) Number of proteins identified across different experiments. Each data point represents an experiment with the number of identified proteins (protein groups). B) Data completeness analysis of DIA TPP-PISA for the DMSO (n=3) and Staurosporine (n=3) experiment used for this analysis. X-axis displays percentage of data which was identified in the different replicates, i.e. >90% of the proteins were identified in 6 of 6 runs (y-axis) for this experiment. Similar data completeness analysis for C) TMT TPP and D) TMT TPP-PISA.

Supplementary Figure 3: 96-well strategy for TPP-PISA experiments

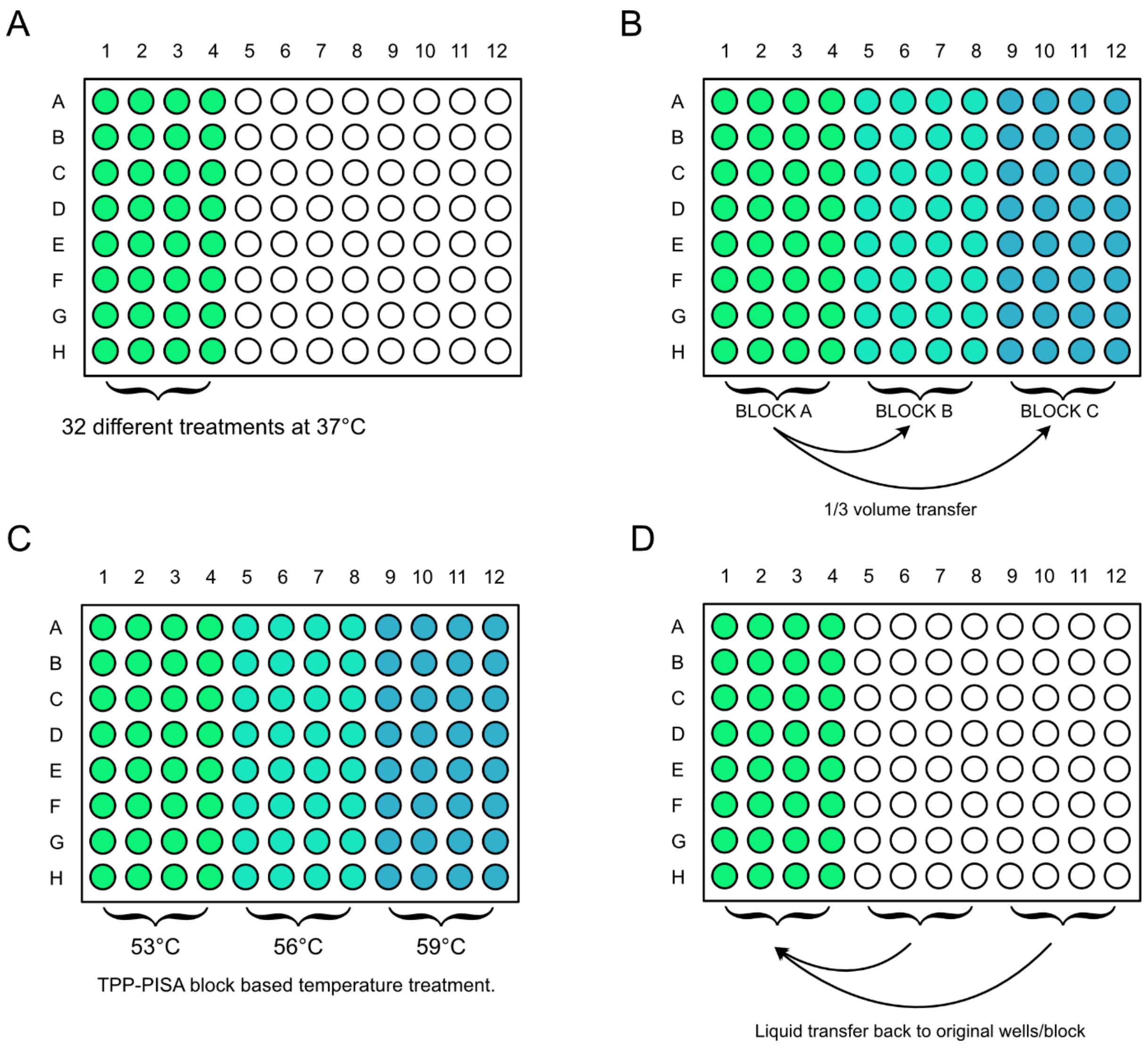

Supplementary Figure 3: The strategy for 96-well format for TPP-PISA experiments which enables up to 32 different treatments in one plate. A) Protein extracts can be aliquoted into rows A-H and columns 1-4 in PCR plates followed by the addition of various drugs and ligands. The mixture is then heated at 37ºC for 10 minutes. B) After experimental treatment, equal volume is distributed to three blocks. This is achieved through transfer of 1/3 of the volume from block A to block B and C, resulting in equal volume in all wells and blocks. C) The blocks are heated at 3 different temperatures as described for the TPP-PISA experiments. D) The liquid volume is transferred back to the original wells and block (block A) from block B and C after temperature treatment.

Supplementary Figure 4: Staurosporine response in Rat organ extracts

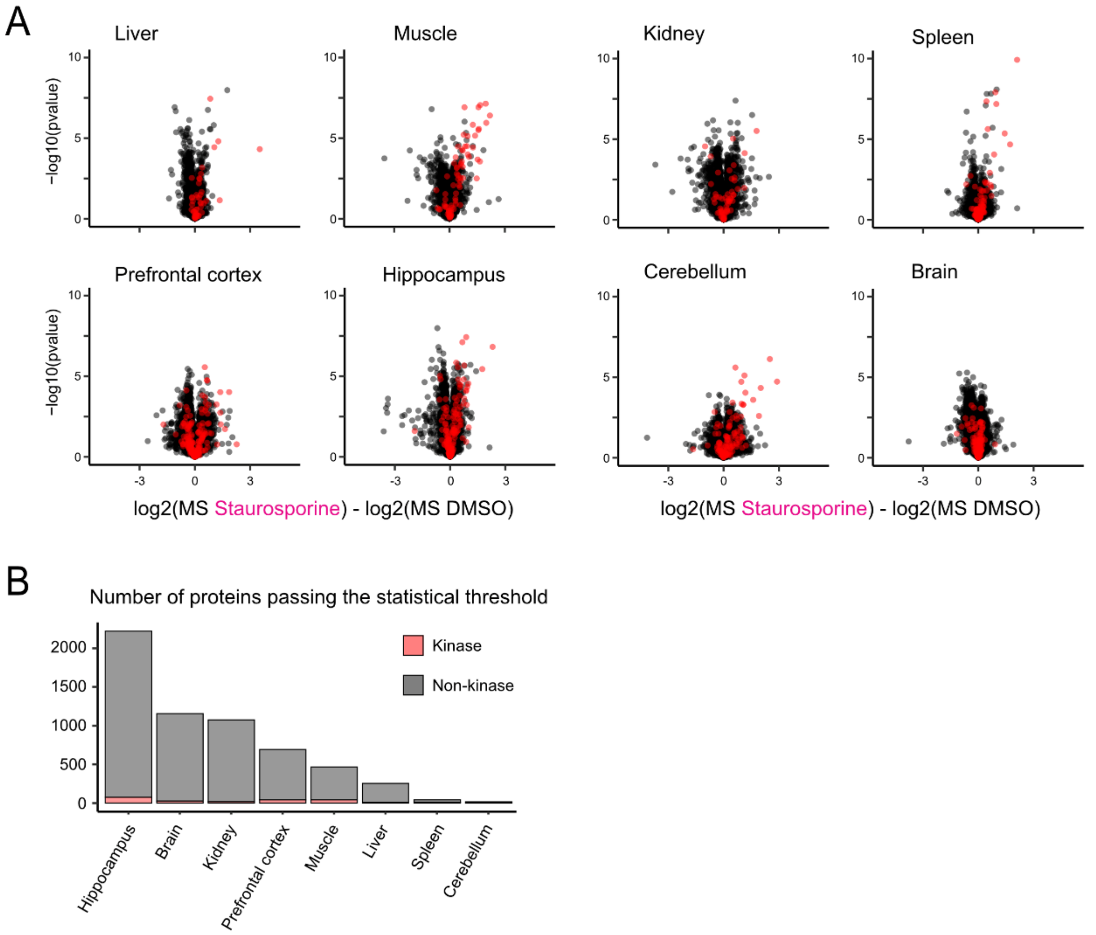

Supplementary Figure 4. A) Volcano plots for all the statistical analysis of rat organs subjected to TPP-PISA with the protocol presented in (A). -log10(p-values) are plotted on the vertical axis, and the differences between log2-transformed protein groups quantities in the soluble fractions of the staurosporine- and DMSO-treated conditions are presented on the horizontal axis. Kinases are highlighted in red. B) Number of hits found in different rat organ extracts with q-value <0.05.

Supplementary Figure 5: Heatmap of Staurosporine targets in Rat organ extracts

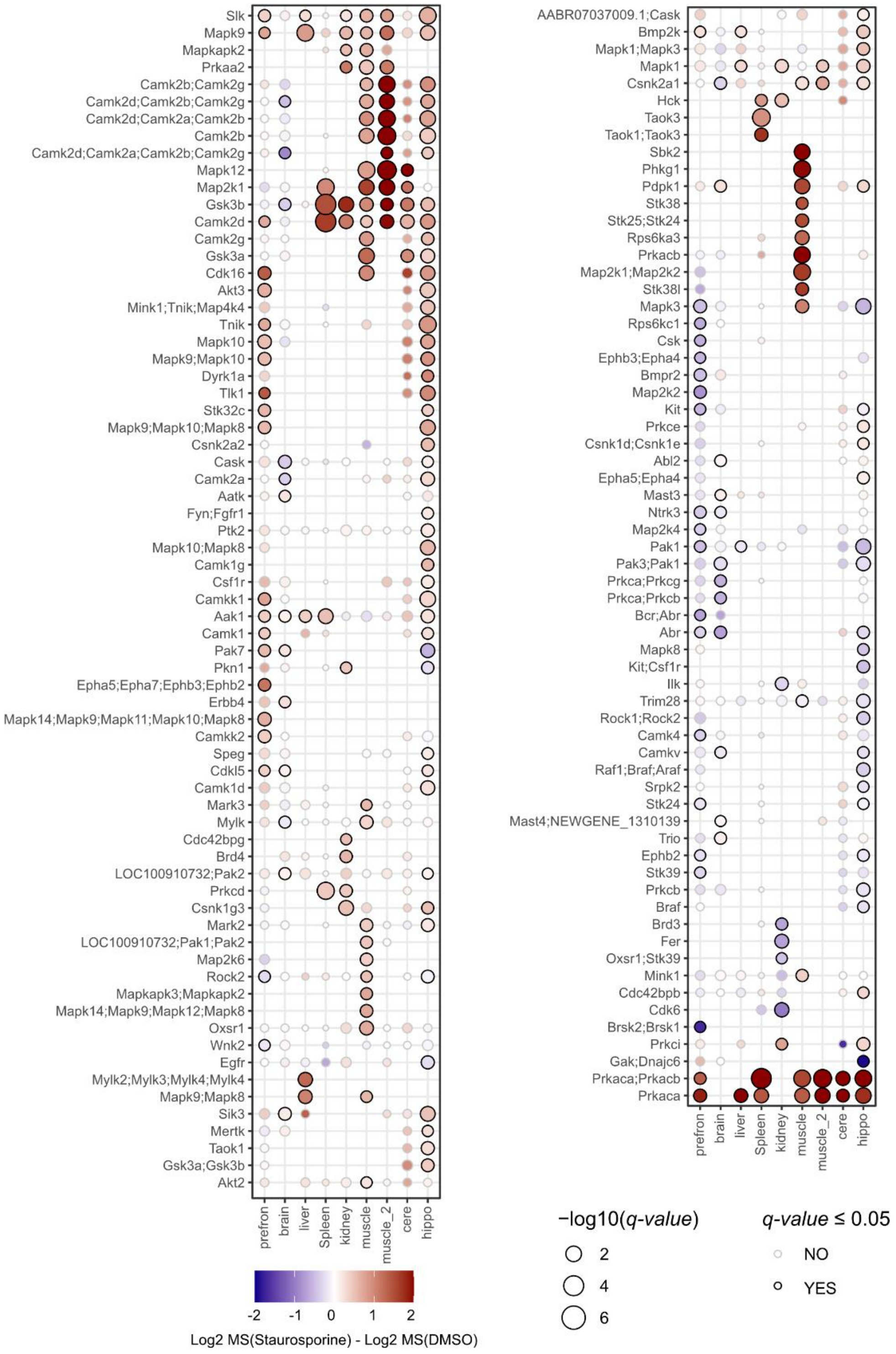

Supplementary Figure 5. Heatmap of all Staurosprorine kinase targets observed in rat organ extracts. Normalized fold change values log2(MS Staurosporine) – log2(MS DMSO) are color coded for each point, and the size of the point reflects the *q-value*. Stroke color for each points displays whether the kinases pass statistical threshold based on fold change and *q-value*.

Supplementary Figure 6: Validation of hPirin positive control inhibitor TPhA by SPR

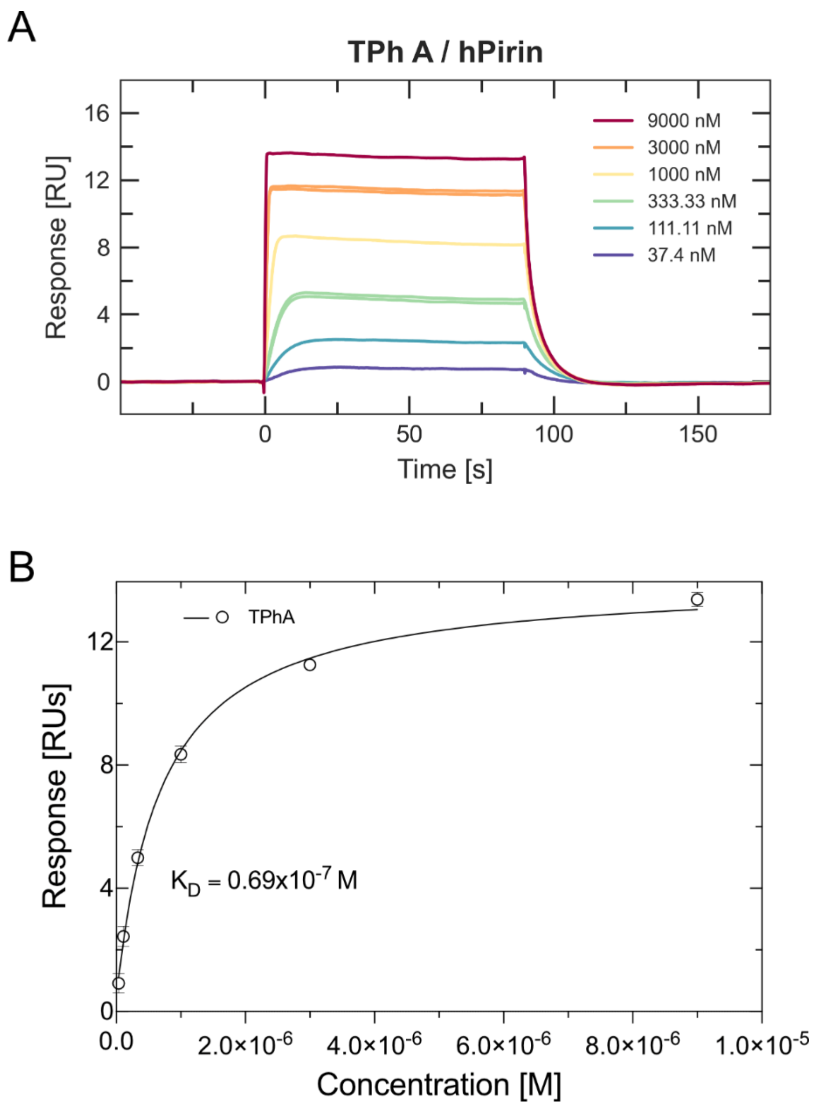

Supplementary Figure 6. **A)** SPR sensorgram of recombinant human Pirin (hPirin) and Triphenyl Compound A (TPhA) showing a dose dependent response. **B)** Equilibrium dissociation constant (Kd) determined from the steady state model of TPhA for hPirin confirming its high specificity.

Supplementary Figure 7: HexB assay in HeLa protein extracts

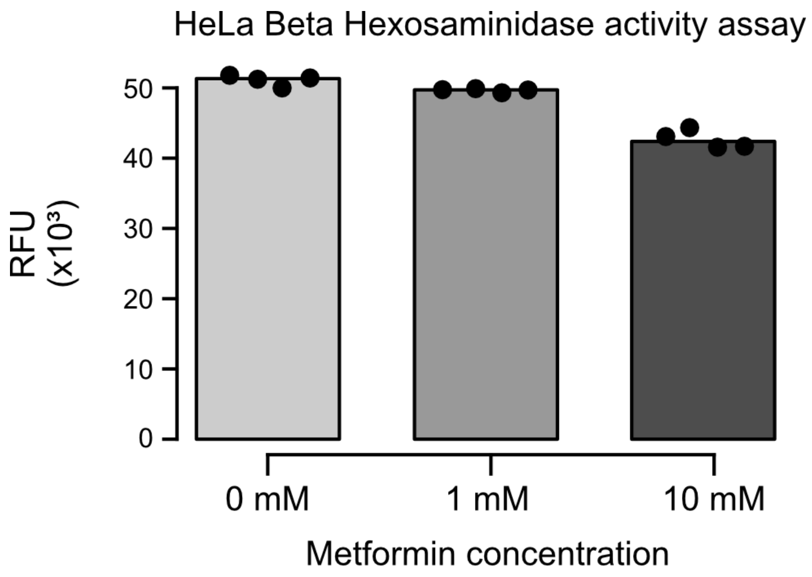

Supplementary Figure 7. HexB activity performed on HeLa cellular extracts using a fluorescent assay with 1mm and 10mm metformin. Relative fluorescence units are plotted. Individual replicates are plotted (n=4).

SUPPLEMENTARY REFERENCES

1. Savitski, M.M., Reinhard, F.B.M., Franken, H., Werner, T., Savitski, M.F., Eberhard, D., Martinez Molina, D., Jafari, R., Dovega, R.B., Klaeger, S., et al. (2014). Tracking cancer drugs in living cells by thermal profiling of the proteome. Science *346*, 1255784. 10.1126/science.1255784.

2. Becher, I., Andrés-Pons, A., Romanov, N., Stein, F., Schramm, M., Baudin, F., Helm, D., Kurzawa, N., Mateus, A., Mackmull, M.-T., et al. (2018). Pervasive Protein Thermal Stability Variation during the Cell Cycle. Cell *173*, 1495-1507.e18. 10.1016/j.cell.2018.03.053.

3. Reinhard, F.B.M., Eberhard, D., Werner, T., Franken, H., Childs, D., Doce, C., Savitski, M.F., Huber, W., Bantscheff, M., Savitski, M.M., et al. (2015). Thermal proteome profiling monitors ligand interactions with cellular membrane proteins. Nat. Methods *12*, 1129–1131. 10.1038/nmeth.3652.
