# Supplementary material for: Streamlined analysis of drug targets by proteome integral solubility alteration indicates organ-specific engagement
Source: Nat Commun. 2024 Oct 16;15:8923. doi: 10.1038/s41467-024-53240-2 (PMC11484808; doi:10.1038/s41467-024-53240-2)
Supplement: Supplementary file 2 — Reporting Summary [file 41467_2024_53240_MOESM2_ESM.pdf]

nature portfolio

Corresponding author(s): Jesper Velgaard Olsen

Last updated by author(s): Sep 27, 2024

# Reporting Summary

Nature Portfolio wishes to improve the reproducibility of the work that we publish. This form provides structure for consistency and transparency in reporting. For further information on Nature Portfolio policies, see our Editorial Policies and the Editorial Policy Checklist.

## Statistics

For all statistical analyses, confirm that the following items are present in the figure legend, table legend, main text, or Methods section.

| n/a | Confirmed | |
|---|---|---|
| ☐ | ☒ | The exact sample size (*n*) for each experimental group/condition, given as a discrete number and unit of measurement |
| ☐ | ☒ | A statement on whether measurements were taken from distinct samples or whether the same sample was measured repeatedly |
| ☐ | ☒ | The statistical test(s) used AND whether they are one- or two-sided<br>*Only common tests should be described solely by name; describe more complex techniques in the Methods section.* |
| ☐ | ☒ | A description of all covariates tested |
| ☐ | ☒ | A description of any assumptions or corrections, such as tests of normality and adjustment for multiple comparisons |
| ☐ | ☒ | A full description of the statistical parameters including central tendency (e.g. means) or other basic estimates (e.g. regression coefficient) AND variation (e.g. standard deviation) or associated estimates of uncertainty (e.g. confidence intervals) |
| ☐ | ☒ | For null hypothesis testing, the test statistic (e.g. *F*, *t*, *r*) with confidence intervals, effect sizes, degrees of freedom and *P* value noted<br>*Give P values as exact values whenever suitable.* |
| ☐ | ☒ | For Bayesian analysis, information on the choice of priors and Markov chain Monte Carlo settings |
| ☐ | ☒ | For hierarchical and complex designs, identification of the appropriate level for tests and full reporting of outcomes |
| ☐ | ☒ | Estimates of effect sizes (e.g. Cohen's *d*, Pearson's *r*), indicating how they were calculated |

*Our web collection on statistics for biologists contains articles on many of the points above.*

## Software and code

Policy information about availability of computer code

| | |
|---|---|
| Data collection | Raw MS data submitted to ProteomeXchange with identifier PXD050784. Reviewer account, Username: reviewer_pxd050784@ebi.ac.uk , Password: 8JGEEWAA |
| Data analysis | Data analysis software available at https://doi.org/10.5281/zenodo.13141610 (DOI 10.5281/zenodo.13141610) |

For manuscripts utilizing custom algorithms or software that are central to the research but not yet described in published literature, software must be made available to editors and reviewers. We strongly encourage code deposition in a community repository (e.g. GitHub). See the Nature Portfolio guidelines for submitting code & software for further information.

## Data

Policy information about availability of data

All manuscripts must include a data availability statement. This statement should provide the following information, where applicable:

- Accession codes, unique identifiers, or web links for publicly available datasets
- A description of any restrictions on data availability
- For clinical datasets or third party data, please ensure that the statement adheres to our policy

The mass spectrometry proteomics data have been deposited to the ProteomeXchange Consortium via the PRIDE partner repository with the dataset identifier PXD050784. Uniprot reference human and rat proteomes were used for database searches.

## Research involving human participants, their data, or biological material

Policy information about studies with human participants or human data. See also policy information about sex, gender (identity/presentation), and sexual orientation and race, ethnicity and racism.

| | |
|---|---|
| Reporting on sex and gender | N/A |
| Reporting on race, ethnicity, or other socially relevant groupings | N/A |
| Population characteristics | N/A |
| Recruitment | N/A |
| Ethics oversight | N/A |

Note that full information on the approval of the study protocol must also be provided in the manuscript.

# Field-specific reporting

Please select the one below that is the best fit for your research. If you are not sure, read the appropriate sections before making your selection.

[x] Life sciences  [ ] Behavioural & social sciences  [ ] Ecological, evolutionary & environmental sciences

For a reference copy of the document with all sections, see nature.com/documents/nr-reporting-summary-flat.pdf

# Life sciences study design

All studies must disclose on these points even when the disclosure is negative.

| | |
|---|---|
| Sample size | No sample size calculation was performed, the number of samples used per experiment were chosen based on previous experience to obtain enough statistical power in DIA experiments. We performed four replicates per experimental condition. |
| Data exclusions | No data was excluded from the analysis. |
| Replication | All experiments were conducted with at least four replicates. |
| Randomization | No randomization was performed for technical benchmark. Clinical samples were randomized before injection. |
| Blinding | Researchers were not blinded to perform the experiments reported in this work.<br>This was required as to keep a strict overview of mass spectrometry method selection. |

# Reporting for specific materials, systems and methods

We require information from authors about some types of materials, experimental systems and methods used in many studies. Here, indicate whether each material, system or method listed is relevant to your study. If you are not sure if a list item applies to your research, read the appropriate section before selecting a response.

## Materials & experimental systems

| n/a | Involved in the study | |
|---|---|---|
| x | | Antibodies |
| | x | Eukaryotic cell lines |
| x | | Palaeontology and archaeology |
| | x | Animals and other organisms |
| x | | Clinical data |
| x | | Dual use research of concern |
| x | | Plants |

## Methods

| n/a | Involved in the study | |
|---|---|---|
| x | | ChIP-seq |
| x | | Flow cytometry |
| x | | MRI-based neuroimaging |

## Eukaryotic cell lines

Policy information about cell lines and Sex and Gender in Research

| | |
|---|---|
| Cell line source(s) | HeLa and HepG2 cells were acquired from ATCC. |
| Authentication | Cell lines were authenticated by STR profiling using the ATCC authentication service according to manufacturers' instructions. |
| Mycoplasma contamination | Cultures tested negative for mycoplasma. |
| Commonly misidentified lines (See ICLAC register) | No commonly misidentified cell lines were employed in this study. |

## Animals and other research organisms

Policy information about studies involving animals; ARRIVE guidelines recommended for reporting animal research, and Sex and Gender in Research

| | |
|---|---|
| Laboratory animals | Adult male Wistar rats (200-220 g at arrival; Charles River, Sulzfeld, Germany) |
| Wild animals | N/A |
| Reporting on sex | Only male subjects were included in the study. |
| Field-collected samples | N/A |
| Ethics oversight | Animal experiments were conducted following the guidelines of the Danish Animal Experimentation Inspectorate (license number: 2016-15-0201-00934) and approved by the local institutional animal care unit. |

Note that full information on the approval of the study protocol must also be provided in the manuscript.

## Plants

| | |
|---|---|
| Seed stocks | N/A |
| Novel plant genotypes | N/A |
| Authentication | N/A |
